# Prediction of Cardiac Remodeling and/or Myocardial Fibrosis Based on Hemodynamic Parameters of Vena Cava in Athletes

**DOI:** 10.2174/0115734056316396241227064057

**Published:** 2025-01-09

**Authors:** Bin-yao Liu, Fan Zhang, Min-song Tang, Xing-yuan Kou, Qian Liu, Xin-rong Fan, Rui Li, Jing Chen

**Affiliations:** 1 Department of Radiology, The Affiliated Hospital of Southwest Medical University, Luzhou, Sichuan, China; 2 Department of Gynaecology and Obstetrics, The Affiliated Hospital of Southwest Medical University, Luzhou, China; 3 Department of Cardiology, The Affiliated Hospital of Southwest Medical University, Luzhou, Sichuan, China; 4 Medical Imaging Key Laboratory of Sichuan Province, North Sichuan Medical College, Nanchong, Sichuan, China, 637000

**Keywords:** 4D flow, Vena cava, Athletes, Machine learning, Cardiovascular adverse events, Myocardial Fibrosis

## Abstract

**Purpose:**

This study aimed to assess the hemodynamic changes in the vena cava and predict the likelihood of Cardiac Remodeling (CR) and Myocardial Fibrosis (MF) in athletes utilizing four-dimensional (4D) parameters.

**Materials and Methods:**

A total of 108 athletes and 29 healthy sedentary controls were prospectively recruited and underwent Cardiac Magnetic Resonance (CMR) scanning. The 4D flow parameters, including both general and advanced parameters of four planes for the Superior Vena Cava (SVC) and Inferior Vena Cava (IVC) (sheets 1-4), were measured and compared between the different groups. Four machine learning models were employed to predict the occurrence of CR and/or MF.

**Results:**

Most general 4D flow parameters related to VC were increased in athletes and positive athletes compared to controls (*p* < 0.05). Gradient Boosting Machine (GBM) was the most effective model in sheet 2 of SVC, with the area under the curve values of 0.891, accuracy of 85.2%, sensitivity of 84.6%, and specificity of 85.4%. The top five predictors in descending order were as follows: net positive volume, forward volume, waist circumference, body weight, and body surface area.

**Conclusion:**

Physical activity can induce a high flow state in the vena cava. CR and/or MF may elevate the peak velocity and maximum pressure gradient of the IVC. This study successfully constructed a GBM model with high efficacy for predicting CR and/or MF. This model may provide guidance on the frequency of follow-up and the development of appropriate exercise plans for athletes.

## 1 INTRODUCTION

The physiological adaptation of the heart induced by strenuous physical exercise is commonly known as the Athlete's Heart (AH). However, this cardiac adaptation exhibits overlapping features with non-ischemic cardiomyopathy and is also associated with Sudden Cardiac Death (SCD) in athletes [1]. Research indicates that athletes face a 2.8-time higher risk of SCD associated with cardiovascular disease compared to non-athletes [2]. Furthermore, cardiovascular-related causes are the leading contributors to sudden death among athletes [3]. Andrélagerge's study revealed a higher prevalence of right ventricular remodeling and Myocardial Fibrosis (MF) in athletes engaged in intense training [4]. MF, often stemming from Cardiac Remodeling (CR), involves a crucial role of pro-inflammatory cytokines in the regulation of initial myocardial remodeling following injury [5]. While CR and MF may lead to adverse cardiovascular events, promising experimental findings demonstrate that some mice with MF, induced by vigorous exercise for 16 weeks, exhibited reversal of the condition after restoring normal physical activity levels for 8 weeks. Additionally, the trend towards severe ventricular arrhythmias had diminished [6]. Therefore, early detection of CR and MF in athletes could significantly mitigate or even reverse cardiac damage, ultimately reducing the risk of SCD and fostering improved cardiovascular outcomes.

Cardiovascular hemodynamics serves as a crucial biomarker in evaluating cardiovascular health. The blood flow of the vena cava, being the inflow channel of the Right Ventricle (RV), profoundly influences the RV's structure and function. Athletes exhibit increased right ventricular lumen size and wall thickness, along with larger inflow and outflow sizes [7, 8]. This observation indicates that exercise progressively enhances venous reflux, resulting in elevated Cardiac Output (CO) in athletes, consequently necessitating a higher oxygen supply for the right ventricular coronary artery. However, during exercise, right ventricular cardiomyocytes may encounter hypoxia, and the abrupt increase in preload can lead to heightened myocardial oxidative stress, damage to the myocardial cell membrane, and endocardial interstitial fibrosis. These factors can contribute to myocardial overstretching and microtears, ultimately resulting in a reduction in myocardial exercise capacity [9]. Prolonged, high-intensity exercise training can induce CR, leading to subsequent chronic changes culminating in MF. The association between the hemodynamic manifestations of the Vena Cava (VC) and CR and MF suggests that alterations in VC blood flow may be closely linked to or potentially precede the development of these cardiac conditions [10]. Consequently, it is plausible to predict early myocardial injury through changes in the blood flow of the VC.

The assessment of VC hemodynamics involves various diagnostic modalities, including Cardiac Magnetic Resonance (CMR), echocardiography, and catheter manometry. Notably, the utilization of four-dimensional (4D) flow CMR is of particular significance, as it enables the acquisition of comprehensive three-dimensional (3D) images and real-time quantification of 3D blood flow in individuals with cardiovascular conditions [11]. This noninvasive technique facilitates the evaluation of blood flow characteristics and offers risk stratification for cardiac diseases [12-15]. 4D flow CMR holds the advantage of allowing repetitive evaluation without the need for invasive procedures or exposure to radiation, and it has demonstrated good consistency with catheterization for VC hemodynamic measurements [16, 17]. Although catheter pressure measurement provides an accurate evaluation of the VC, its invasive nature renders it less favorable for routine clinical practice. In this context, non-invasive 4D flow CMR emerges as an invaluable tool for the hemodynamic assessment of the VC, offering a safer and more patient-friendly approach.

The existing literature has predominantly focused on investigating the correlation between the diameter of the VC and right ventricular function in athletes [18]. However, there is a paucity of research examining the hemodynamics of the VC in this population, particularly in relation to predicting CR and MF, which may hold greater prognostic value than solely assessing existing adverse cardiovascular events. Therefore, this study aimed to comprehensively evaluate exercise-induced changes in VC hemodynamics using advanced 4D flow CMR technology. Additionally, the study developed prediction models based on these VC hemodynamic parameters using Machine Learning (ML) techniques, renowned for their efficacy in predicting CR and/or MF. These models may guide the frequency of athlete follow-up and potentially reduce the incidence of adverse remodeling and fibrosis.

## 2 MATERIALS AND METHODS

In accordance with the Declaration of Helsinki (2013), this study received approval from the institutional review committee of our hospital (reference number: KY2020123). All participants provided voluntary and informed consent through a signed agreement.

In accordance with established sports standards [19], this study prospectively recruited 125 athletes from May 2020 to January 2023. The inclusion criteria were as follows: (1) continuous participation in sports for more than 3 years, with a weekly sports engagement exceeding 6 hours (accompanied by a comprehensive sports record); (2) demonstration of good general health, including the absence of heart disease (such as hypertrophic cardiomyopathy, dilated cardiomyopathy, left ventricular noncompaction, arrhythmogenic right ventricular cardiomyopathy, *etc*.) and cardiovascular risk factors; (3) no contraindications to CMR, such as metal implants in the body, early pregnancy, claustrophobia, or other conditions. The exclusion criteria were as follows: (1) cessation of exercise for more than 6 months; (2) poor CMR image quality impeding post-processing; (3) use of certain drugs, such as muscle-boosting substances. Ultimately, a total of 108 exercisers [98 males and 9 females; median age, 24.0 years (22.0; 27.0)] were included in the study. Additionally, 29 healthy sedentary controls (23 males and 6 females; median age, 23.0 years (23.0; 25.0)) were recruited, exhibiting no abnormal results in relevant physical examinations. The control group reported exercising for no more than 3 hours per week, with no regular cumulative exercise time.

Before the examination, researchers conducted a comprehensive assessment of all participants, including measurements of height, weight, blood pressure, heart rate, neck circumference, waist circumference, and an Electrocardiogram (ECG). They also recorded relevant information regarding smoking history, alcohol consumption history, and family medical history. The study required all subjects to abstain from vigorous exercise, coffee, or alcohol within 24 hours before the examination. Participants were informed about the CMR examination process, its purpose, and potential risks. All volunteers provided voluntary informed consent for the CMR scanning procedure.

### 2.1 CMR Parameters

All examinations were performed using a 3.0T CMR system (Siemens Healthcare, Prisma, Erlangen, Germany). The cardiac cine scanning utilized Steady-state Free Precession (SSFP) breath-hold sequences in chambers 2, 3, and 4, and 8-12-layer continuous short-axis images. The scanning parameters were as follows: repetition time (TR) = 66.2 ms, echo time (TE) = 1.46 ms, layer thickness = 6 mm, layer spacing = 1 mm, Field of View (FOV) = 340 mm, voxel size = 1.5 × 1.5 × 6.0 mm^3^.

For 4D flow CMR examinations, sagittal three-dimensional volumes covering the caval veins were acquired during free breathing. The acquisition duration varied between 8 to 15 minutes based on the heart rate, gated to the cardiac cycle using the ECG signal. A series of time-resolved (cine) images were obtained to capture the dynamics of pulsatile blood flow throughout the cardiac cycle. The scanning parameters were as follows: velocity encoding = 80 cm/s, TE = 2.88 ms, TR = 45.36 ms, echo spacing = 5.7 ms, spatial resolution = 1.8 × 1.8 × 3.5 mm^3^. Phase-sensitive Inversion Recovery (PSIR) sequences for delayed enhancement images were acquired 10 minutes following the administration of gadolinium contrast agent (Shanghai, China, 0.1 mmol/kg). The parameters were as follows: TR = 926.4 ms, TE = 1.24 ms, echo spacing = 3.0 ms, and slice thickness = 6 mm.

### 2.2 Image Analysis

**Fig. (1):**
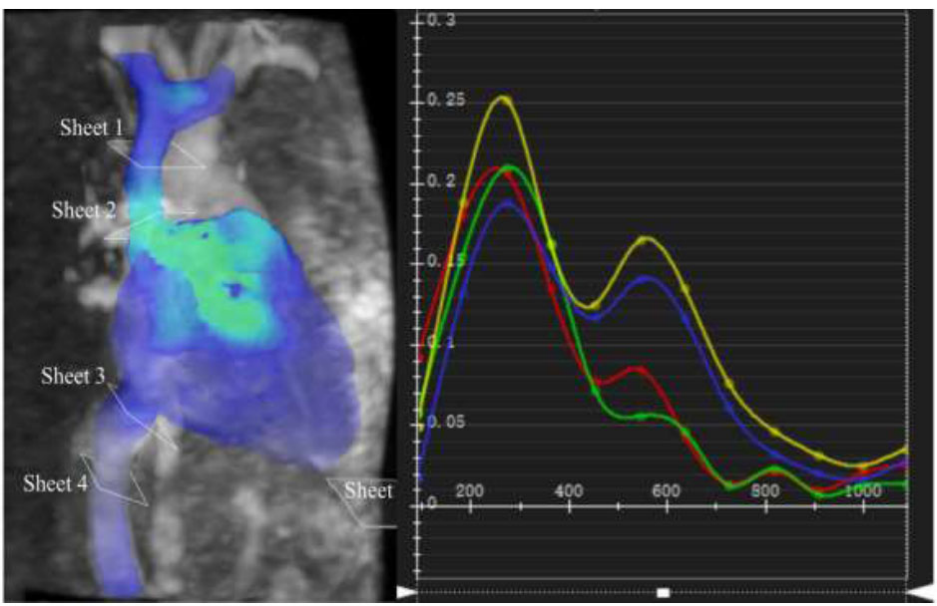
The analysis planes of vena cava using 4D flow CMRI. The two analysis planes were set separately in the superior and inferior vena cava, including the planes below the bifurcation of the superior vena cava into right brachiocephalic and left brachiocephalic branches, at the emptying of the superior vena cava into the right atrium, at the inferior vena cava distal to above the diaphragm, and at the distal branch of hepatic vein.

All acquired images were processed using the CVI 42 software (version 5.12.4, Circle Vascular Imaging, Calgary, Canada) by two experienced radiologists, each having more than 3 years of expertise in cardiovascular CMR imaging. The post-processing procedure involved importing the 4D flow image into the corresponding processing module, and the software automatically recognized the VC contour with semi-automation. Subsequently, the radiologists verified the accuracy of the contour and made manual corrections to ensure precise delineation of the VC contour. Two analysis planes were set separately in the Superior Vena Cava (SVC) and Inferior Vena Cava (IVC) [20], including the planes below the bifurcation of the SVC into right brachiocephalic and left brachiocephalic branches (sheet 1), at the emptying of the SVC into the right atrium (sheet 2), at the IVC distal to above the diaphragm (sheet 3), and at the distal branch of the hepatic vein (sheet 4) (Fig. **1**).

The CVI 42 software automatically calculated both the structural and functional parameters of the VC using the defined analysis planes. Hemodynamic parameters, encompassing general and advanced metrics, were computed by manually positioning a total of four two-dimensional analysis planes on specific anatomic landmarks. The general parameters included forward volume, backward volume, net positive volume, net negative volume, peak velocity, maximum pressure gradient, and maximum flow. The advanced parameters comprised Wall Shear Stress (WSS), Relative Pressure (RP), and Energy Loss (EL).

The cine sequence images were processed following well-established and standardized guidelines [21]. The delineated parameters of the Left Ventricle (LV) and Right Ventricle (RV) were automatically obtained, including End-diastolic Volume (EDV), End-systolic Volume (ESV), Ejection Fraction (EF), CO, Cardiac Index (CI), Stroke Volume (SV), myocardial mass, and the ratio of the aforementioned parameters to Body Surface Area (BSA) and height.

In the study, two experienced radiologists independently analyzed the delayed enhancement images to identify the presence of Myocardial Delayed Enhancement (MDE) in the LV and RV, characterized by increased myocardial signal intensity. The radiologists further described the location, shape, and type of MDE, including linear, patchy, subendocardial, or transmural patterns. In cases of disagreement between the radiologists, they discussed their observations to reach a consensus opinion. Additionally, at the end-diastolic four-chamber view, the study measured the atrial and ventricular long and short diameters, as well as the thickness of the interventricular septum and ventricular free wall. These measurements, combined with cardiac function parameters exceeding the 99th percentile upper reference limit, were considered indicative of CR [22-23]. Athletes with CR and/or MDE were designated as the positive athlete group, while those without were categorized as the negative athlete group.

Intra-observer variability was assessed by comparing measurements from a single observer across 20 randomized cases at 4-week intervals. Interobserver variability was evaluated by two independent, experienced observers in a double-blind manner.

### 2.3 Statistical Analysis

This study employed R software (R Core Team 2020, Vienna, Austria) for data analysis. Intra-group Correlation Coefficients (ICCs) were utilized to evaluate the repeatability and assess intra- and inter-observer variability. Subsequent statistical analyses were conducted when ICCs were greater than 0.8, indicating good repeatability. The normality of the data was tested, and accordingly, results have been presented as “mean ± standard deviation” for normally distributed data and as “median” for data with skewed distribution. To compare the hemodynamic parameters of VC and basic clinical data between different groups (athletes *vs*. controls, positive athlete group *vs*. negative athlete group), the Chi-square test, independent samples t-test, and Mann-Whitney U-test were employed. All the above comparisons were defined as statistically significant by *p* < 0.05. Given the limited number of athletes with MDE, and considering that both CR and MDE could lead to adverse cardiovascular events in athletes, CR and/or MDE were chosen as the outcome variables. Dimension reduction for these basic and 4D flow parameters was performed using the aforementioned methods between positive and negative athlete groups. Subsequently, four ML methods were used to predict the occurrence of CR and/or MDE, including Gradient Boosting Machine (GBM), Logistic Regression (LR), Classification and Regression Tree (CART), and Support Vector Machine (SVM). The selection of these four models considered various factors, such as complexity, performance, interpretability, computational efficiency, and the ability to handle noise, suitable for the medical data and problem in this study. Specifically, the study utilized GBM for its excellent discrimination performance, SVM for handling high-dimensional data, LR for its ability to explain variable-binary outcome relationships, and CART for its effectiveness in handling variable types and interactions [24, 25]. The R package (DALEX) was interpreted for the above four ML methods, and residual distribution was plotted to identify the best model. Receiver Operating Characteristic (ROC) curves and the corresponding Area Under the Curve (AUC), accuracy, sensitivity, and specificity were plotted to compare the performance of the four ML methods. Decision Curve Analysis (DCA) was used to quantify the clinical utility of the prediction models.

## 3 RESULTS

### 3.1 Basic Clinical Data

Table **S1** presents the basic characteristics of the athlete group and control group. MDE was detected in 4 male athletes, with one case located in the inferior wall of the basal segment of the LV and three cases in the basal segment of the left ventricular septal wall (shape of line, less than 2%), and there were 25 male athletes with CR. In the athlete group, many cardiac parameters, including EDV, ESV, SV, CO, and CI of both ventricles, were significantly higher compared to the control group (*p* < 0.05) Table **S2**).

### 3.2 Comparison of 4D Flow Parameters of VC between Different Groups

Tables **1** and **2** display the comparison of meaningful parameters between different groups, while the less significant parameters are presented in Tables **S3** and **S4**. At sheet 1 and sheet 2 levels of the SVC, the athlete group exhibited significantly higher forward volume, maximum flow, and net positive volume compared to the controls (*p* < 0.001). Furthermore, at the sheet 2 level of the SVC, the net negative volume was greater in the athlete group than in the controls (*p* < 0.05). At sheet 3 and sheet 4 levels of the IVC, the athlete group demonstrated greater backward volume and net negative volume than the controls (*p* < 0.05). Additionally, at the sheet 3 level of the IVC, the peak velocity, net positive volume, and maximum pressure gradient were smaller in the athlete group compared to the controls (*p* < 0.05). However, no statistically significant differences were observed between the athlete group and the controls regarding the advanced parameters of both the SVC and IVC. Similarly, there were no significant differences between the positive athlete group with CR and/or MF and the negative athlete group (*p* > 0.05).

The positive group exhibited significantly higher values compared to the negative group (*p* <0.05) across several parameters: forward volume (sheet 1, 2), net positive volume (sheet 1, 2), backward volume (sheet 4), peak velocity, and maximum pressure gradient (sheet 4).

### 3.3 Machine Learning-based Prediction Models for CR and/or MDE

**Fig. (2):**
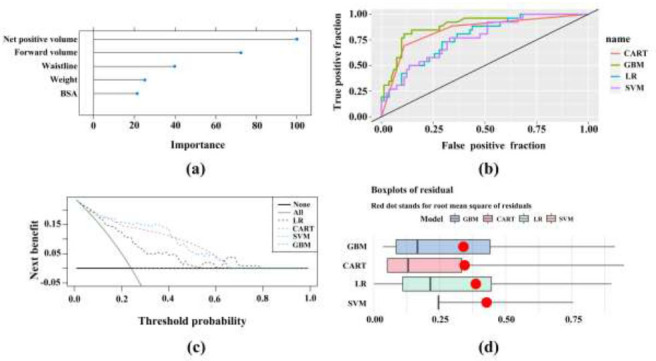
The predictive capabilities of four machine learning models of sheet 2. (**a**) The prediction factors ranked by importance in GBM. (**b**) The Receiver Operating Characteristics (ROC) curves of all four machine learning models (CART, GBM, LR, SVM). The GBM model demonstrated the highest Area Under the Curve (AUC). (**c**) The decision curve map of the CART, GBM, LR, and SVM models, indicating that all four prediction models had a net clinical benefit. (**d**) The boxplot of the residual values of all four machine learning models. The boxplot analysis of residual values for the four prediction models indicated a favorable goodness-of-fit.

The four machine learning models (GBM, SVM, LR, and CART) demonstrated promising predictive capabilities within the validation set at sheet 2 of the SVC (Fig. **2**). Among these models, GBM exhibited the highest effectiveness in predicting CR and/or MDE, with an AUC value of 0.891, accuracy of 85.2%, sensitivity of 84.6%, and specificity of 85.4%. The results from the GBM model indicated the relative importance ranking of predictors. The top five predictors, in descending order, were identified as follows: net positive volume, forward volume, waist circumference, body weight, and BSA.

### 3.4 Reproductivity

The intra-observer variability of 4D flow parameters of VC ranged from 0.810 to 0.933, while the inter-observer variability ranged from 0.818 to 0.932.

## 4 DISCUSSION

### 4.1 Main Findings

Exercise can induce a high-flow state in the VC. CR and/or MF may increase the peak velocity and maximum pressure gradient of the IVC compared to non-remodeling athletes. This study successfully constructed a GBM model to predict CR and/or MF in athletes, which may be associated with adverse cardiovascular events. The model could provide a reference for athletes to appropriately adjust follow-up frequency and exercise intensity using non-invasive 4D flow CMR. Among the four levels of the VC, the level at the emptying of the SVC into the right atrium was found to be the most sensitive to sport-induced CR. During follow-up, particular attention should be given to net positive volume, forward volume, waist circumference, body weight, and BSA at the SVC's emptying into the right atrium, especially net positive volume.

### 4.2 The Hemodynamic Characteristics of VC in Athletes

This study found that athletes had a significantly higher volume of VC return blood compared to the control group. This finding can be explained by the increased oxygen demand during exercise, which leads to an increase in CO and, subsequently, an increase in VC blood volume. Exercise-induced remodeling includes physiological cardiomegaly as a key component [26]. However, prolonged pressure or volume overload may cause further remodeling, characterized by ventricular dilation [27, 28]. The enlargement of the LV results in an increase in CO and venous blood volume within the VC. Similarly, the RV expands to optimize venous return collection [10]. Nevertheless, an increase in venous reflux exacerbates CR and/or MF. Elevated CO increases right ventricular systolic pressure, amplifying the stress on the right ventricular wall, resulting in increased afterload and work. As a result, this situation significantly increases coronary artery perfusion and oxygen demand for the RV, potentially causing hypoxia during exercise. A sudden increase in preload during exercise may lead to heightened oxidative stress and damage to right ventricular cardiomyocytes, triggering the release of biomarkers of myocardial injury, initiating an immune cell response, and resulting in endocardial interstitial fibrosis and perivascular fibrous tissue proliferation. These processes cause excessive stretching and micro-tearing of the myocardium, leading to a decrease in myocardial motility [9]. Prolonged and high-intensity exercise training can cause repeated damage to cardiac muscle cells, leading to CR, and subsequent chronic changes can ultimately result in MF [10]. This also confirms the observation in our study that athletes with CR and/or MF had a greater volume of VC return blood.

In the athletes' group, the peak velocity and maximum pressure gradient at the sheet 3 level for the IVC were observed to be smaller compared to the control group. These parameters are closely associated with the size and blood flow of the vascular lumen, suggesting that the reduction in blood vessel diameter and blood flow could account for the decrease in peak velocity and maximum pressure gradient [8]. Vascular endothelial cells, residing in the innermost layer of the blood vessel wall, play a pivotal role in regulating vascular relaxation and contraction [29, 30]. Among the factors produced by these cells, Endothelin-1 (ET-1) exerts robust vasoconstrictor and proliferative activities on vascular smooth muscle cells [31]. Notably, studies have indicated that strength training athletes exhibit increased ET-1 concentration in their plasma [32], likely attributed to the significant rise in blood pressure during exercise. The elevated ET-1 levels may stimulate the thickening of the vascular wall, safeguarding it from the impact of intensified blood pressure during strenuous exercise. Furthermore, the vasoconstrictor property of ET-1 can lead to a reduced diameter of the VC, consequently contributing to the observed decline in peak velocity and maximum pressure gradient [32]. Surprisingly, at the sheet 4 level of IVC, the peak velocity and maximum pressure gradient in positive athletes were higher than those in negative athletes. This disparity can potentially be attributed to the beneficial effects of appropriate exercise on improving vascular endothelial cell function. However, long-term and high-intensity exercise may inflict damage to endothelial cells. In cases where endothelial cells sustain excessive injuries due to sports-related trauma, their capacity to regulate vasomotor tension becomes compromised. This results in the expansion of the VC diameter in response to increased blood flow, subsequently leading to higher peak velocity and maximum pressure gradient in positive athletes compared to negative athletes. Notably, healthy endothelial cells are crucial for maintaining hemostasis and preventing thrombosis. Occasionally, cases of athlete venous thrombosis have been reported, indirectly indicating that excessive exercise can potentially harm endothelial cells.

### 4.3 Clinical Application of the GBM Model

Engaging in extensive periods of high-intensity exercise training can lead to CR, potentially resulting in MF, which frequently manifests as MDE [33]. This progression may provide a foundation for sudden cardiac arrhythmia and even SCD in athletes [2]. In addition to the risk of arrhythmia, MF can cause increased myocardial stiffness and localized cardiac dysfunction [34].

The gradual increase in venous return flow induced by exercise may contribute to the development of CR and MF as a consequence of the resulting elevation in return flow and subsequent myocardial cell hypoxia [9]. Therefore, it is crucial to proactively predict the occurrence of CR and/or MF in athletes based on the observed hemodynamic alterations in the VC. Such early identification of these conditions may be valuable in protecting the well-being of athletes.

The GBM model for sheet 2 of the SVC revealed net positive volume and forward volume to be the two most influential variables, suggesting their increased sensitivity to exercise-induced chronic CR and MF. This finding can be explained from two perspectives. First, the lower heart rate observed in athletes could prolong the cardiac cycle, particularly during diastole [35-37]. As a result, the time required for venous blood to return to the heart increases, leading to an increase in the net positive volume and forward volume of the VC. Second, when athletes experience right ventricular remodeling, the heart cavity expands [38]. This expansion increases the internal negative pressure, enhancing the heart's ability to reserve blood flow and further contributing to the elevation of net positive volume and forward volume in the VC, especially at the proximal end of the SVC (sheet 2). Importantly, since the blood flow in the SVC closely resembles that of the IVC, the SVC experiences early effects during the short cardiac cycle of blood pumping. Monitoring the net positive volume and forward volume on sheet 2 of the SVC can be crucial in determining appropriate follow-up frequencies for athletes.

The GBM model has demonstrated the significance of BSA, body weight, and waist circumference as variables of high relative importance. The presence of individual differences necessitates considering the weight and BSA of subjects, as these factors better reflect each individual's unique characteristics. A prior study investigating the relationship between body size and survival in patients with chronic heart failure resulting from left ventricular systolic dysfunction found BSA to be the most potent single predictor. Patients with larger BSA exhibited higher survival rates, with weight and waist circumference also emerging as notable predictors [39]. Overweight status and increased waist circumference lead to greater visceral fat accumulation, which is associated with various cardiovascular risk factors [40], inflammatory and oxidative stress markers [41], liver steatosis [42], and atherosclerosis [43]. The importance of these screened variables suggests the validity of the GBM model. However, we did not use the factors to predict CR and MF, but instead validated the effectiveness of our model using the selected effective predictive factors. Our model can be used to screen high-risk patients, and these predictive indicators may have a definite association with outcome variables involved in cardiovascular disease.

### 4.4 Limitations

The current study was constrained by its single-center dataset. To enhance the robustness of the machine learning model, future work will incorporate data from multiple centers for further validation. Moreover, the number of positive cases among the athletes was relatively small, partially due to the limited range of exercise loads and lacking higher-intensity activities, such as triathlons. Additionally, individual differences and genetic factors among the athletes may have influenced the occurrence of CR and MF. During the one-year follow-up period, no adverse cardiovascular events were reported for these athletes, potentially associated with the increased attention and vigilance provided through frequent monitoring. Nevertheless, the follow-up process will continue to validate and reinforce these findings over an extended period. Lastly, invasive puncture examinations were not performed to distinguish pathological from physiological remodeling, as some pathological remodeling may lack clinical symptoms in the early stages, potentially leading to suboptimal puncture results. Previous studies have demonstrated ventricular expansion and myocardial thickening to increase the probability of adverse events, regardless of the underlying cause.

## CONCLUSION

Exercise-induced alterations in Vena Cava (VC) hemodynamics can result in a high-flow state within the VC. CR and/or MF could elevate the peak velocity and maximum pressure gradient of the IVC. Given the observed correlation between VC hemodynamics and the presence of CR and MF, we have successfully developed a GBM model based on VC hemodynamic parameters to predict the likelihood of CR and/or MF occurrence in athletes. As these conditions may be associated with adverse cardiovascular events, their early prediction could be beneficial for adjusting exercise frequency or promptly detecting potential lesions. During future follow-up processes, we recommend focusing on monitoring the net positive volume and forward volume at the emptying of the SVC into the right atrium.

## Figures and Tables

**Table 1:** Comparison of 4D flow parameters between the athletes and the control volunteers.

-	-	Total (n = 137)	Athletes (n = 108)	Controls (n = 23)	*P*-values
Forward volume (ml)	Sheet 1	30.0± 8.8	32.1± 8.2	22.0 ± 5.5	<0.001
-	Sheet 2	26.0±6.7	27.1±6.8	21.8±4.3	< 0.001
Backward volume (ml)	Sheet 3	-45.3 ± 17.7	-47.6 ± 16.8	-36.8 ± 18.4	0.003
-	Sheet 4	-38.9 ± 15.3	-41.9 ± 14.6	-27.7 ± 12.8	< 0.001
Peak velocity (cm/s)	Sheet 3	56.3 (46.0, 67.5)	55.2 (44.9, 64.8)	62.4 (53.1, 73.0)	0.034
Max pressure gradient (mmHg)	Sheet 3	1.3 (0.9, 1.8)	1.2 (0.8, 1.7)	1.6 (1.1, 2.1)	0.036
Net positive volume (ml)	Sheet 1	32.0(25.9,39.0)	33.8(28.3,40.5)	24.4(21.0,30.6)	<0.001
-	Sheet 2	27.4(23.4,32.1)	28.7(24.5,33.0)	24.1(19.7,27.3)	< 0.001
-	Sheet 3	1.2 (0.4, 2.3)	1 (0.4, 2.1)	1.7 (0.8, 3.0)	0.035
Net negative volume (ml)	Sheet 2	1.7 (0.9,3.1)	1.8 (1.1,3.2)	1.2 (0.5,2.1)	0.036
-	Sheet 3	-46.1 ± 17.6	-48.1 ± 17	-38.5 ± 18.0	0.008
-	Sheet 4	-39.3 ± 15.6	-42.5 ± 14.6	-27.5 ± 13.8	< 0.001
RPmax (mmHg)	IVC	-0.3 (-0.6, 0)	-0.3 (-0.7, -0.1)	0 (-0.4, 0.2)	0.005

**Note: **Values are mean ± SD or median (Q1, Q3) or n (%). Values in bold indicate significant differences between groups. SVC, superior vena cava; IVC, inferior vena cava; WSSmax, maximum wall shear stress; WSSavg, average wall shear stress; RPmax, maximum relative pressure; RPavg, average relative pressure; ELmax, maximum viscous energy loss; ELavg, average viscous energy loss.

**Table 2:** Comparison of 4D flow parameters between negative and positive athletes with CR and/or MF.

-	-	Total (n = 137)	Athletes (n = 108)	Controls (n = 23)	*P*-values
Forward volume (ml)	Sheet 1	32.1 ± 8.2	30.7 ± 7.5	36.6 ± 8.7	0.004
-	Sheet 2	27.1 ± 6.8	25.9 ± 6.8	30.9 ± 5.3	< 0.001
Backward volume (ml)	Sheet 4	-41.9 ± 14.6	-39.7 ± 12.8	-48.7 ± 17.8	0.023
Peak velocity (cm/s)	Sheet 4	68.2 (52.5, 89.5)	64.1 (51.8, 86.7)	79 (67.2, 102.6)	0.033
Max pressure gradient (mmHg)	Sheet 4	1.9 (1.1, 3.2)	1.6 (1.1, 3)	2.5 (1.8, 4.2)	0.033
Net positive volume (ml)	Sheet 1	33.8 (28.6, 40.2)	32.5 (27.2, 36.2)	39.9 (32.6, 44.7)	0.001
-	Sheet 2	28.6 ± 7.2	27.4 ± 7.2	32.7 ± 5.8	< 0.001

**Note: **Values are mean ± SD or Mmedian (Q1, Q3) or n (%). Values in bold indicate significant differences between groups. SVC, superior vena cava; IVC, inferior vena cava; WSSmax, maximum wall shear stress; WSSavg, average wall shear stress; RPmax, maximum relative pressure; RPavg, average relative pressure; ELmax, maximum viscous energy loss; ELavg, average viscous energy loss.

## Data Availability

The data and supportive information is available within the article.

## LIST OF ABBREVIATIONS

CR: Cardiac Remodeling
MF: Myocardial Fibrosis
GBM: Gradient Boosting Machine
AH: Athlete's Heart
SCD: Sudden Cardiac Death
RV: Right Ventricle
VC: Vena Cava
CMR: Cardiac Magnetic Resonance
ECG: Electrocardiogram
SSFP: Steady-state Free Precession
FOV: Field of View
PSIR: Phase-sensitive Inversion Recovery
IVC: Inferior Vena Cava
SVC: Superior Vena Cava
WSS: Wall Shear Stress
LV: Left Ventricle
RV: Right Ventricle
EDV: End-diastolic Volume
ESV: End-systolic Volume
EF: Ejection Fraction
CI: Cardiac Index
SV: Stroke Volume
BSA: Body Surface Area
MDE: Myocardial Delayed Enhancement
ICCs: Intra-group Correlation Coefficients
GBM: Gradient Boosting Machine
LR: Logistic Regression
CART: Classification and Regression Tree
SVM: Support Vector Machine
DCA: Decision Curve Analysis

## STANDARDS OF REPORTING

STROBE guidelines were followed.

## SUPPLEMENTARY MATERIAL

Supplementray material is available on the publisher's website along with the published article.

## References

[1] Szabo L., Brunetti G., Cipriani A., Juhasz V., Graziano F., Hirschberg K., Dohy Z., Balla D., Drobni Z., Perazzolo Marra M., Corrado D., Merkely B., Zorzi A., Vago H. (2022). Certainties and uncertainties of cardiac magnetic resonance imaging in Athletes.. J. Cardiovasc. Dev. Dis..

[2] Corrado D., Basso C., Rizzoli G., Schiavon M., Thiene G. (2003). Does sports activity enhance the risk of sudden death in adolescents and young adults?. J. Am. Coll. Cardiol..

[3] Maron B.J., Haas T.S., Murphy C.J., Ahluwalia A., Rutten-Ramos S. (2014). Incidence and causes of sudden death in U.S. college athletes.. J. Am. Coll. Cardiol..

[4] La Gerche A., Burns A.T., Mooney D.J., Inder W.J., Taylor A.J., Bogaert J., MacIsaac A.I., Heidbüchel H., Prior D.L. (2012). Exercise-induced right ventricular dysfunction and structural remodelling in endurance athletes.. Eur. Heart J..

[5] Oka R., Miura K., Sakurai M., Nakamura K., Yagi K., Miyamoto S., Moriuchi T., Mabuchi H., Koizumi J., Nomura H., Takeda Y., Inazu A., Nohara A., Kawashiri M.A., Nagasawa S., Kobayashi J., Yamagishi M. (2010). Impacts of visceral adipose tissue and subcutaneous adipose tissue on metabolic risk factors in middle-aged Japanese.. Obesity (Silver Spring).

[6] Benito B., Gay-Jordi G., Serrano-Mollar A., Guasch E., Shi Y., Tardif J.C., Brugada J., Nattel S., Mont L. (2011). Cardiac arrhythmogenic remodeling in a rat model of longterm intensive exercise training.. Circulation.

[7] Oxborough D., Sharma S., Shave R., Whyte G., Birch K., Artis N., Batterham A.M., George K. (2012). The right ventricle of the endurance athlete: The relationship between morphology and deformation.. J. Am. Soc. Echocardiogr..

[8] Forsythe L., Somauroo J., George K., Papadakis M., Brown B., Qasem M., Oxborough D. (2019). The right heart of the elite senior rugby football league athlete.. Echocardiography.

[9] Schaafs L.A., Tzschätzsch H., Figiel C., van der Giet M., Reshetnik A., Hamm B., Sack I., Elgeti T. (2019). Quantitative time-harmonic ultrasound elastography of the abdominal aorta and inferior vena cava.. Ultrasound Med. Biol..

[10] Cecchi E., Giglioli C., Valente S., Lazzeri C., Gensini G.F., Abbate R., Mannini L. (2011). Role of hemodynamic shear stress in cardiovascular disease.. Atherosclerosis.

[11] Nerem R.M. (1992). Vascular fluid mechanics, the arterial wall, and atherosclerosis.. J. Biomech. Eng..

[12] Markl M., Frydrychowicz A., Kozerke S., Hope M., Wieben O. (2012). 4D flow MRI.. J. Magn. Reson. Imaging.

[13] Erol M.K., Karakelleoglu S. (2002). Assessment of right heart function in the athlete’s heart.. Heart Vessels.

[14] Onizuka H., Sueyoshi E., Sakamoto I., Miura T. (2017). Dilation of inferior vena cava and iliac veins in elite athlete.. J. Vasc. Surg. Venous Lymphat. Disord..

[15] Rahman O., Markl M., Balte P., Berhane H., Blanken C., Suwa K., Dashnaw S., Wieben O., Bluemke D.A., Prince M.R., Lima J., Michos E., Ambale-Venkatesh B., Hoffman E.A., Gomes A.S., Watson K., Sun Y., Carr J., Barr R.G. (2019). Reproducibility and changes in vena caval blood flow by using 4D flow MRI in pulmonary emphysema and chronic obstructive pulmonary disease (COPD): The Multi-ethnic study of atherosclerosis (MESA) COPD substudy.. Radiology.

[16] Dyverfeldt P, Bissell M, Barker AJ (2015). 4D flow cardiovascular magnetic resonance consensus statement.. J Cardiovasc Magn Reson.

[17] Fernandes JF, Gill H, Nio A (2023). Non-invasive cardiovascular magnetic resonance assessment of pressure recovery distance after aortic valve stenosis.. J Cardiovasc Magn Reson.

[18] Lei X., Liu H., Han Y., Cheng W., Sun J., Luo Y., Yang D., Dong Y., Chung Y., Chen Y. (2017). Reference values of cardiac ventricular structure and function by steady-state free-procession MRI at 3.0T in healthy adult chinese volunteers.. J. Magn. Reson. Imaging.

[19] Aquaro G.D., Camastra G., Monti L., Lombardi M., Pepe A., Castelletti S., Maestrini V., Todiere G., Masci P., di Giovine G., Barison A., Dellegrottaglie S., Perazzolo Marra M., Pontone G., Di Bella G., working group “Applicazioni della Risonanza Magnetica” of the Italian Society of Cardiology (2017). Reference values of cardiac volumes, dimensions, and new functional parameters by MR: A multicenter, multivendor study.. J. Magn. Reson. Imaging.

[20] Li W, Wan K, Han Y (2017). Reference value of left and right atrial size and phasic function by SSFP CMR at 3.0T in healthy Chinese adults. Sci Rep.

[21] Ori Y., Korzets A., Katz M., Perek Y., Zahavi I., Gafter U. (1996). Haemodialysis arteriovenous access: A prospective haemodynamic evaluation.. Nephrol. Dial. Transplant..

[22] MacRae J.M., Pandeya S., Humen D.P., Krivitski N., Lindsay R.M. (2004). Arteriovenous fistula-associated high-output cardiac failure: A review of mechanisms.. Am. J. Kidney Dis..

[23] Langer S., Paulus N., Heiss C., Koeppel T.A., Greiner A., Buhl A., Lauer T., Kokozidou M., Jacobs M.J., Krombach G.A., European Vascular Center Aachen-Maastricht (2011). Cardiovascular remodeling after AVF surgery in rats assessed by a clinical MRI scanner.. Magn. Reson. Imaging.

[24] Shehab M., Abualigah L., Shambour Q., Abu-Hashem M.A., Shambour M.K.Y., Alsalibi A.I., Gandomi A.H. (2022). Machine learning in medical applications: A review of state-of-the-art methods.. Comput. Biol. Med..

[25] Li J, Zhu Y, Dong Z (2022). Development and validation of a feature extraction-based logical anthropomorphic diagnostic system for early gastric cancer: A case-control study.. E Clinical Med.

[26] Pluim B.M., Zwinderman A.H., van der Laarse A., van der Wall E.E. (2000). The athlete’s heart. A meta-analysis of cardiac structure and function.. Circulation.

[27] Qasem M., George K., Somauroo J., Forsythe L., Brown B., Oxborough D. (2019). Right ventricular function in elite male athletes meeting the structural echocardiographic task force criteria for arrhythmogenic right ventricular cardiomyopathy.. J. Sports Sci..

[28] Merlo M., Gobbo M., Artico J., Sinagra G., Merlo M., Pinamonti B. (2019). Etiological definition and diagnostic work-up.. Dilated Cardiomyopathy: From Genetics to Clinical Management..

[29] Weeks K.L., McMullen J.R. (2011). The athlete’s heart *vs*. the failing heart: Can signaling explain the two distinct outcomes?. Physiology (Bethesda).

[30] Cheng C.P., Herfkens R.J., Taylor C.A. (2003). Inferior vena caval hemodynamics quantified *in vivo* at rest and during cycling exercise using magnetic resonance imaging.. Am. J. Physiol. Heart Circ. Physiol..

[31] van de Schoor F.R., Aengevaeren V.L., Hopman M.T.E., Oxborough D.L., George K.P., Thompson P.D., Eijsvogels T.M.H. (2016). Myocardial fibrosis in athletes.. Mayo Clin. Proc..

[32] Dooley S., ten Dijke P. (2012). TGF-β in progression of liver disease.. Cell Tissue Res..

[33] Shimizu I., Minamino T. (2016). Physiological and pathological cardiac hypertrophy.. J. Mol. Cell. Cardiol..

[34] Hernandez-Gea V., Friedman S.L. (2011). Pathogenesis of liver fibrosis.. Annu. Rev. Pathol..

[35] Moretti R., Pizzi B. (2010). Inferior vena cava distensibility as a predictor of fluid responsiveness in patients with subarachnoid hemorrhage.. Neurocrit. Care.

[36] Song Y, Jia H, Hua Y (2022). The molecular mechanism of aerobic exercise improving vascular remodeling in hypertension.. Front Physiol.

[37] Heusch G. (2008). Heart rate in the pathophysiology of coronary blood flow and myocardial ischaemia: Benefit from selective bradycardic agents.. Br. J. Pharmacol..

[38] Palmisano A., Darvizeh F., Cundari G., Rovere G., Ferrandino G., Nicoletti V., Cilia F., De Vizio S., Palumbo R., Esposito A., Francone M. (2021). Advanced cardiac imaging in athlete’s heart: Unravelling the grey zone between physiologic adaptation and pathology.. Radiol. Med. (Torino).

[39] Futter J.E., Cleland J.G.F., Clark A.L. (2011). Body mass indices and outcome in patients with chronic heart failure.. Eur. J. Heart Fail..

[40] Fox C.S., Massaro J.M., Hoffmann U., Pou K.M., Maurovich-Horvat P., Liu C.Y., Vasan R.S., Murabito J.M., Meigs J.B., Cupples L.A., D’Agostino R.B., O’Donnell C.J. (2007). Abdominal visceral and subcutaneous adipose tissue compartments: Association with metabolic risk factors in the Framingham heart study.. Circulation.

[41] Pou K.M., Massaro J.M., Hoffmann U., Vasan R.S., Maurovich-Horvat P., Larson M.G., Keaney J.F., Meigs J.B., Lipinska I., Kathiresan S., Murabito J.M., O’Donnell C.J., Benjamin E.J., Fox C.S. (2007). Visceral and subcutaneous adipose tissue volumes are cross-sectionally related to markers of inflammation and oxidative stress: The Framingham heart study.. Circulation.

[42] Ducluzeau P.H., Manchec-Poilblanc P., Roullier V., Cesbron E., Lebigot J., Bertrais S., Aubé C. (2010). Distribution of abdominal adipose tissue as a predictor of hepatic steatosis assessed by MRI.. Clin. Radiol..

[43] Landsberg L., Young J.B. (1978). Fasting, feeding and regulation of the sympathetic nervous system.. N. Engl. J. Med..

