# Supplementary material for: Prediction of Cardiac Remodeling and/or Myocardial Fibrosis Based on Hemodynamic Parameters of Vena Cava in Athletes
Source: Curr Med Imaging. 2025 Jan 9;21:e15734056316396. doi: 10.2174/0115734056316396241227064057 (PMC12933233; doi:10.2174/0115734056316396241227064057)
Supplement: Supplementary file 1 [file CMIM-21-E15734056316396_SD1.pdf]

1573-4056/25 *Send Orders for Reprints to reprints@benthamscience.net*

BENTHAM SCIENCE
CrossMark

Current Medical Imaging

Content list available at: https://benthamscience.com/journals/cmir

Supplementary Material

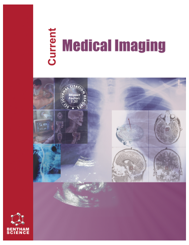

Prediction of Cardiac Remodeling and/or Myocardial Fibrosis Based on Hemodynamic Parameters of Vena Cava in Athletes

Bin-yao Liu[1,#], Fan Zhang[2,#], Min-song Tang[1], Xing-yuan Kou[1], Qian Liu[1], Xin-rong Fan[3], Rui Li[4] and Jing Chen[1,*]

[1]*Department of Radiology, The Affiliated Hospital of Southwest Medical University, Luzhou, Sichuan, China*
[2]*Department of Gynaecology and Ostetrics, The Affiliated Hospital of Southwest Medical University, Luzhou, China*
[3]*Department of Cardiology, The Affiliated Hospital of Southwest Medical University, Luzhou, Sichuan, China*
[4]*Medical Imaging Key Laboratory of Sichuan Province, North Sichuan Medical College, Nanchong, Sichuan, China, 637000*

Table S1. Comparison of basic clinical characteristics between the athletes and the control volunteers.

-	**Total (n = 137)**	**Athletes (n = 108)**	**Controls (n = 23)**	***p* values**
Age (yrs)	24 (22, 27)	24 (22, 27)	23 (23, 25)	0.69
Height (cm)	173 (167, 176)	173 (168, 176)	170 (160, 176)	0.124
Weight (kg)	67.6 ± 10.0	68.6 ± 9.3	60.8 ± 12.2	**0.04**
BMI (kg/m^2)	22.5 (20.9, 24.4)	22.7 (21.4, 24.6)	20.7 (18.7, 23.5)	**0.009**
BSA (m^2)	1.7 ± 0.2	1.8 ± 0.2	1.7 ± 0.2	0.1
Neck circumference (cm)	36 (34, 37.9)	36.5 (35, 38)	34 (30.3, 37)	**0.008**
waistline (cm)	80.9 ± 8.0	81.5 ± 7.8	76.3 ± 8.5	0.19
SBP (mmHg)	119.1 ± 13	119.5 ± 13.1	116.5 ± 11.6	0.4
DBP (mmHg)	74 (67.8, 80.3)	74 (69, 80)	69 (62, 84)	0.213
Heart rate at rest (beats/min)	61(56, 67)	61(56, 66)	65(59, 68)	0.092

Note: Values are Mean ± SD or Median (Q1,Q3) or n (%). Values in bold indicate significant differences between groups.. BMI, body mass index; BSA, body surface area; SBP, systolic blood pressure; DBP, diastolic blood pressure.

Table S2. Comparison of cardiac function parameters between the athletes and the control volunteers.

-	**Total (n = 137)**	**Athletes (n = 108)**	**Controls (n = 23)**	***p* values**
RVEDV (ml)	180.3 ± 32	184.5 ± 30.7	150.5 ±24.9	**< 0.001**
RVESV (ml)	89.3 ± 18,4	90.9 ± 17.9	77.4 ± 18.1	**0.007**
RVSV (ml)	90.1 (76.6, 102.3)	90.9 (79.7, 104)	71.3 (65.3, 82.4)	**< 0.001**
RVEF (n%)	50.8 (46.0, 54.8)	51.1 (46.7, 55)	46 (43.6, 54)	0.201
RVCO(L/min)	5.5(4.8, 6.4)	5.6(5.0, 6.4)	4.3(3.7, 5.6)	**0.003**
RVCI (L/min/m^2)	3.1 ± 0.6	3.1 ± 0.6	2.9 ± 0.6	0.13
RVEDV/BSA (ml/m^2)	100.6 ± 14.0	101.8 ± 14.1	91.6 ± 10.2	**0.008**
RVESV/BSA (ml/m^2)	48.2(43.6, 56.5)	48.2(44.4, 56.4)	47.9(40.2, 56.8)	0.38
RVSV/BSA (ml/m^2)	50.6 ± 9.2	51.5 ± 9.3	44.4 ± 5.5	**< 0.001**
LVEDV (ml)	161.7 ± 28.1	166.2 ± 25.8	129.2 ± 22.3	**< 0.001**
LVESV (ml)	66 ± 15.2	68.1 ± 14.2	51.7 ± 15.0	**< 0.001**
LVSV (ml)	95.6 ± 18.8	98.2 ± 18	77.6 ± 14.1	**< 0.001**

DOI: 10.2174/0115734056316396241227064057, 2025, 21, e15734056316396

-	Total (n = 137)	Athletes (n = 108)	Controls (n = 23)	*p* values
LVEF (n%)	59.2 ± 5.7	59.1 ± 5.5	60.3 ± 6.9	0.422
LVCO (L/min)	5.9(5.1, 6.6)	6.0(5.2, 6.7)	4.6(4.3, 5.8)	**0.001**
LVCI (L/min/m^2)	3.3(2.9, 3.6)	3.3(2.9, 3.6)	3.0(2.7, 3.2)	0.042
LVEDV/BSA (ml/m^2)	90.1 ± 12.2	91.7 ± 11.8	78.6 ± 9.0	**< 0.001**
LVESV/BSA (ml/m^2)	36.7(31.8, 41.9)	38.1(32.4, 42.2)	30.4.1(25.1, 33.4)	**0.002**
LVSV/BSA (ml/m^2)	53.3 ± 8.6	54.2 ± 8.6	47.2 ± 5.8	**0.003**

Note: Values are Mean ± SD or Median (Q1,Q3) or n (%). Values in bold indicate significant differences between groups. RVEDV, right ventricular end-diastolic volume; RVESV, right ventricular end-systolic volume; RVSV, right ventricular stroke volume; RVEF, right ventricular ejection fraction; RVCO, right ventricular cardiac output; RVCI, right ventricular cardiac index; LVEDV, left ventricular end-diastolic volume; LVESV, left ventricular end-systolic volume; LVSV, left ventricular stroke volume; LVEF, left ventricular ejection fraction; LVCO, left ventricular cardiac output; LVCI, left ventricular cardiac index.

Table S3. Comparison of 4D flow parameters between the athletes and the control volunteers.

-	-	Total (n = 137)	Athletes (n = 108)	Controls (n = 23)	*p* values
Forward Volume(ml)	Sheet 3	0 (0, 0.3)	0 (0, 0.2)	0 (0, 1.0)	0.113
-	Sheet 4	0 (0, 0.2)	0 (0, 0.2)	0 (0, 0.2)	0.43
Backward Volume(ml)	Sheet 1	-0.1(-0.6,0)	-0.1(-0.6,0)	-0.1(-0.6,0)	0.414
-	Sheet 2	-0.4 (-0.9, 0)	-0.5 (-1.0, 0)	-0.2 (-0.8, 0)	0.266
Peak Velocity(cm/s)	Sheet 1	71.4(58.5,85.5)	70.4(58.4,80.8)	82.3(58.5,100.0)	0.081
-	Sheet 2	55.7 (45.9, 76.9)	56.0 (45.8, 75.7)	55.7 (46.2, 88.2	0.602
-	Sheet 4	68.0 (51.6, 90.3)	68.2 (52.3, 89.6)	67.8 (45.5, 97.7)	0.7
Max Pressure Gradient(mmHg)	Sheet 1	2.0(1.3,2.9)	2.0(1.4,2.6)	2.7(1.4,2.0)	0.081
-	Sheet 2	1.2 (0.8, 2.4)	1.3 (0.8, 2.3)	1.2 (0.9, 3.1)	0.602
-	Sheet 4	1.8 (1.1, 3.3)	1.9 (1.1, 3.2)	1.8 (0.8, 3.8)	0.7
Net Positive Volume(ml)	Sheet 4	0.5 (0.2, 1.4)	0.5 (0.2, 1.3)	0.5 (0.4, 2.4)	0.258
Net Negative Volume(ml)	Sheet 1	2.2(1.0,4.0)	2.2(1.1,3.8)	2.5(0.7,5.6)	0.891
WSSmax (N/m2)	Sheet 1	0.2(0.2,0.3)	0.2(0.2,0.3)	0.2(0.2,0.2)	0.29
-	Sheet 2	0.2 (0.2, 0.2)	0.2 (0.2, 0.2)	0.2 (0.1, 0.2)	0.122
-	Sheet 3	0.2 (0.1, 0.2)	0.2 (0.1, 0.2)	0.2 (0.1, 0.3)	0.733
-	Sheet 4	0.3 ± 0.1	0.3 ± 0.1	0.2 ± 0.1	0.105
WSSavg (N/m2)	Sheet 1	0.1(0.1,0.1)	0.1(0.1,0.1)	0.1(0.1,0.1)	0.487
-	Sheet 2	0.1 ± 0	0.1 ± 0	0.1 ± 0	0.777
-	Sheet 3	0.1 ± 0	0.1 ± 0	0.1 ± 0	0.532
-	Sheet 4	0.1 (0.1, 0.2)	0.2 (0.1, 0.2)	0.1 (0.1, 0.2)	0.302
RPmax (mmHg)	SVC	1.1 (0.7, 2.0)	1.2 (0.8, 2.1)	1.0 (0.5, 2.6)	0.08
RPavg (mmHg)	SVC	1.8 (1.3, 3.0)	1.8 (1.3, 2.9)	2.0 (1.0, 3.2)	0.931
-	IVC	-0.2 (-0.7, 0.1)	-0.3 (-0.7, 0.1)	-0.2 (-0.9, 0.1)	0.649
ELmax (mW)	SVC	0.3(0.2,0.4)	0.3(0.2,0.4)	0.3(0.2,0.4)	0.118
-	IVC	0.3 (0.2, 0.5)	0.3 (0.2, 0.5)	0.2 (0.1, 0.4)	0.117
ELavg (mW)	SVC	0.1(0.1,0.1)	0.1(0.1,0.1)	0.1(0.1,0.1)	0.136
-	IVC	0.2 (0.1, 0.3)	0.2 (0.1, 0.3)	0.1 (0.1, 0.2)	0.093

Note: Values are Mean ± SD or Median (Q1,Q3) or n (%). Values in bold indicate significant differences between groups.SVC,superior vena cava;IVC,inferior vena cava;WSSmax, maximum wall shear stress; WSSavg, average wall shear stress; RPmax, maximum relative pressure; RPavg, average relative pressure; ELmax, maximum viscous energy loss; ELavg, average viscous energy loss.

Table S4. Comparison of 4D flow parameters between negative and positive athletes with CR and/or MF.

-	-	Total (n = 137)	Athletes (n = 108)	Controls (n = 23)	*p* values
Forward Volume(ml)	Sheet 3	0 (0, 0.2)	0 (0, 0.2)	0 (0, 0.3)	0.958
-	Sheet 4	0 (0, 0.2)	0 (0, 0.2)	0 (0, 0.5)	0.789
Backward Volume(ml)	Sheet 1	-0.1 (-0.6, 0)	-0.1 (-0.5, 0)	-0.2 (-0.8, 0)	0.315
-	Sheet 2	-0.5 (-0.9, 0)	-0.4 (-0.9, 0)	-0.7 (-1.6, -0.2)	0.271
-	Sheet 3	-47.6 ± 16.8	-45.9 ± 15.7	-53.1 ± 19.3	0.091
Peak Velocity(cm/s)	Sheet 1	70.4 (58.5, 80.7)	67.8 (57.5, 80.1)	72.9 (64, 84.2)	0.118
-	Sheet 2	56 (45.9, 75.5)	54.7 (45.6, 73.1)	64.1 (50.1, 85.5)	0.134

-	-	Total (n = 137)	Athletes (n = 108)	Controls (n = 23)	*p* values
-	Sheet 3	55.2 (44.9, 63.9)	53.8 (43.1, 64.8)	56.1 (48.8, 61.9)	0.475
Max Pressure Gradient(mmHg)	Sheet 1	2 (1.4, 2.6)	1.8 (1.3, 2.6)	2.1 (1.6, 2.8)	0.118
-	Sheet 2	1.3 (0.8, 2.3)	1.2 (0.8, 2.1)	1.6 (1, 2.9)	0.133
-	Sheet 3	1.2 (0.8, 1.6)	1.2 (0.7, 1.7)	1.3 (1, 1.5)	0.339
Net Positive Volume(ml)	Sheet 3	1 (0.4, 2.1)	0.9 (0.4, 2.1)	1.1 (0.5, 2.1)	0.744
-	Sheet 4	0.5 (0.2, 1.2)	0.5 (0.2, 1.2)	0.6 (0.2, 1.3)	0.774
Net Negative Volume(ml)	Sheet 1	-2.2 (-3.8, -1.1)	-2 (-3.7, -1.1)	-2.9 (-4.3, -1.3)	0.249
-	Sheet 2	-1.8 (-3.1, -1.1)	-1.7 (-2.6, -0.9)	-2.2 (-3.8, -1.4)	0.058
-	Sheet 3	-48.1 ± 17	-46.2 ± 15.8	-54.3 ± 19.3	0.058
-	Sheet 4	-41.4 (-49.3, -33)	-39.8 (-47.8, -33)	-47.5 (-61.2, -33.1)	0.075
WSSmax (N/m2)	Sheet 1	0.2 (0.2, 0.3)	0.2 (0.2, 0.3)	0.2 (0.2, 0.2)	0.302
-	Sheet 2	0.2 ± 0	0.2 ± 0	0.2 ± 0	0.339
-	Sheet 3	0.2 ± 0.1	0.2 ± 0.1	0.2 ± 0.1	0.627
-	Sheet 4	0.3 ± 0.1	0.3 ± 0.1	0.3 ± 0.1	0.674
WSSavg (N/m2)	Sheet 1	0.1 (0.1, 0.1)	0.1 (0.1, 0.1)	0.1 (0.1, 0.1)	0.502
-	Sheet 2	0.1 ± 0	0.1 ± 0	0.1 ± 0	0.621
-	Sheet 3	0.1 ± 0	0.1 ± 0	0.1 ± 0	0.753
-	Sheet 4	0.2 (0.1, 0.2)	0.1 (0.1, 0.2)	0.2 (0.1, 0.2)	0.703
RPmax (mmHg)	SVC	1.2 (0.8, 2)	1.2 (0.9, 2.1)	0.9 (0.6, 1.6)	0.176
-	IVC	1.8 (1.3, 2.8)	1.9 (1.3, 2.9)	1.8 (1.4, 2.7)	1
RPavg (mmHg)	SVC	-0.3 (-0.7, -0.1)	-0.4 (-0.7, -0.1)	-0.2 (-0.6, 0)	0.33
-	IVC	-0.3 (-0.7, 0.1)	-0.2 (-0.7, 0.1)	-0.3 (-0.6, 0.2)	0.824
ELmax (mW)	SVC	0.3 (0.2, 0.4)	0.3 (0.2, 0.4)	0.3 (0.1, 0.5)	0.541
-	IVC	0.3 (0.2, 0.5)	0.3 (0.2, 0.5)	0.4 (0.2, 0.5)	0.209
ELavg (mW)	SVC	0.1 (0.1, 0.1)	0.1 (0.1, 0.1)	0.1 (0.1, 0.1)	0.36
-	IVC	0.2 (0.1, 0.3)	0.2 (0.1, 0.3)	0.2 (0.1, 0.3)	0.247

Note: Values are Mean ± SD or Median (Q1,Q3) or n (%). Values in bold indicate significant differences between groups.SVC,superior vena cava;IVC,inferior vena cava;WSSmax, maximum wall shear stress; WSSavg, average wall shear stress; RPmax, maximum relative pressure; RPavg, average relative pressure; ELmax, maximum viscous energy loss; ELavg, average viscous energy loss.

© 2025 The Author(s). Published by Bentham Science Publisher.

This is an open access article distributed under the terms of the Creative Commons Attribution 4.0 International Public License (CC-BY 4.0), a copy of which is available at: https://creativecommons.org/licenses/by/4.0/legalcode. This license permits unrestricted use, distribution, and reproduction in any medium, provided the original author and source are credited.
